# Thyroid cancer: experience at a state pediatric referral center

**DOI:** 10.1590/1806-9282.20252217

**Published:** 2026-07-10

**Authors:** Denise Bousfield da Silva, Larissa Cubas, Jefferson Traebert, Marilza Leal Nascimento

**Affiliations:** 1 Universidade Federal de Santa Catarina, Department of Pediatrics – Florianópolis (SC), Brazil.; 2 Universidade do Sul de Santa Catarina, Postgraduate Program in Health Sciences – Palhoça (SC), Brazil.; 3 Family and Community Physician for the Municipal Government – Blumenau (SC), Brazil.

**Keywords:** Thyroid neoplasms, Child, Adolescent

## Abstract

**OBJECTIVE:**

The aim of this study was to describe the clinical-epidemiological profile and outcome in patients under 15 years of age with differentiated thyroid cancer diagnosed at a state pediatric referral service in Santa Catarina, Brazil, from 2011 to 2020.

**METHODS:**

Retrospective cohort study analyzing the medical records of 16 patients with differentiated thyroid cancer. The variables analyzed were gender, age, year of diagnosis, previous exposure to radiation, personal and family medical history, symptoms/signs at diagnosis, histological type, staging, therapy, surgical complications, remission rate, and vital status.

**RESULTS:**

Differentiated thyroid cancer occurred in females and adolescents in 62.5% of cases, respectively. Previous radiotherapy was reported in 25% of cases. On physical examination, 56% had a thyroid nodule. Papillary carcinoma was diagnosed in 81.25% of cases. Four patients (25%) were classified as high risk and three never achieving remission. All five low-risk patients and four of the five intermediate-risk patients were in remission. Total thyroidectomy, with or without lymph node resection, was indicated in all cases. Transient hypoparathyroidism occurred in 25%. Radioiodine therapy with I-131 was used in 68.75% of cases, including two low-risk patients treated before 2015. The remission rate was 75%. There were no deaths.

**CONCLUSION:**

Papillary carcinoma is the most common histological type, predominantly in adolescents and females. Previous radiotherapy is a risk factor for differentiated thyroid cancer. The most frequent presentation is a thyroid nodule. Total thyroidectomy is the standard procedure. Radioiodine therapy is not indicated for low-risk patients after 2015. Transient hypoparathyroidism is the most common surgical complication. The remission rate for differentiated thyroid cancer is high.

## INTRODUCTION

Thyroid cancer (TC) is a rare disease in children and adolescents, with an incidence of approximately one case per million per year in children under 10 years old and 18 cases per million in adolescents^1^. It is the most common endocrine cancer in children and adolescents^2^.

In children, the risk of malignancy in thyroid nodules is substantially higher, ranging from 22 to 26%, in contrast to the rate observed in adults, which is 5–10%^1,2–6^. Pediatric patients tend to have more advanced disease at diagnosis, including a higher frequency of lymph node involvement, distant metastasis, and multifocality^4^.

In 2015, the American Thyroid Association (ATA) published guidelines for the management of pediatric TC, recommending changes in the indications for radioiodine therapy (I-131), which is no longer universally encouraged due to the potential increase in malignancy risk and the lack of evidence demonstrating benefit in all cases^3^.

In this context, investigations focused on TC in children and adolescents are essential to support more individualized practices and improve long-term clinical outcomes.

Thus, the objective of this study is to describe the demographic profile, clinical characteristics, and outcomes of a retrospective cohort of pediatric patients with TC treated at a state referral center over a 10-year period.

## METHODS

This retrospective cohort study included all patients under the age of 15 (n=16) with histopathologically confirmed TC who were followed up at a state pediatric referral service in Santa Catarina, Brazil, from 2011 to 2020.

The study was approved by the Brazilian Human Research Ethics Committee (REC), CAAE:35894820.2.0000.5361.

The variables analyzed were gender, age at diagnosis, year of diagnosis, personal medical history, symptoms and signs at diagnosis, histological type, staging, therapy, surgical complications, disease remission rate, and vital status.

The Tumor-Node-Metastasis (TNM) system of the American Joint Committee on Cancer, eighth edition, was used for disease staging^7^. Patients were classified into low, intermediate, or high-risk groups according to the ATA stratification^2,3^.

Before 2015, radioiodine therapy was indicated for all patients with papillary thyroid carcinoma (PTC), and afterward, it was used only for patients with intermediate or high risk^3^.

Radioiodine therapy (I-131) was used only after thyroid-stimulating hormone (TSH) levels reached above 30 mIU/L^3^.

To assess disease remission, thyroglobulin (Tg) levels, the presence of anti-thyroglobulin antibodies (anti-Tg), and imaging tests were considered on the date of the last follow-up. Cases of medullary thyroid carcinoma (MTC) were monitored for calcitonin levels.

The statistical procedures used were descriptive measures and frequency tables. Microsoft Excel (version 2021) was employed for data organization and handling.

## RESULTS

This retrospective cohort study included all patients under the age of 15 (n=16) with histopathologically confirmed TC.

At diagnosis, the mean age was 10.59±3.26 years, and the median was 10.92 years (interquartile range of 7.92 to 13.29 years).

They were adolescents in 62.5% of cases, and 62.5% were female, with a female/male ratio of 1.7:1.

**Figure 1:**
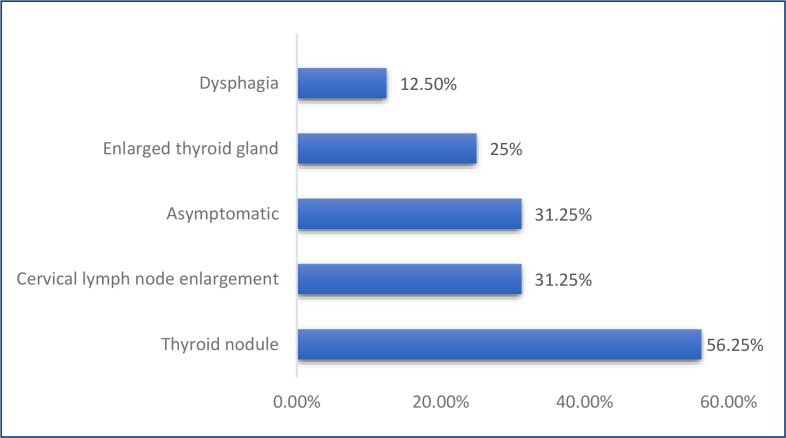
Figure 1. Main signs and symptoms at diagnosis of the 16 cases of thyroid cancer treated at a state pediatric referral service in Santa Catarina, Brazil, from January 2011 to December 2020.

An isolated thyroid nodule was the most frequent clinical presentation (Figure 1). Five patients were asymptomatic, with the suspected diagnosis made based on cervical ultrasound findings or a family history of Multiple Endocrine Neoplasia (MEN) 2. There was no family history of thyroid malignancy.

A quarter of the patients had a history of previous cancer with radiotherapy in the cervical region, including three cases of Hodgkin lymphoma and one case of medulloblastoma.

Hypothyroidism was present in three cases. The pathogenic RET gene variant (codon 634, p.Cys634Arg) was confirmed in both cases with a family history of MEN2.

Total thyroidectomy (TT) was the surgical treatment used in all cases, associated with lymph node resection in 11 (Table 1). Transient hypoparathyroidism occurred in 25% of cases, and one patient had permanent vocal cord paralysis as a complication of surgery.

**Table 1:** Main variables of the 16 cases of thyroid cancer treated at a state pediatric referral service in Santa Catarina, Brazil, from January 2011 to December 2020.

Year of diagnosis	Age at diagnosis	Sex	Histology	Previous history	Surgery	Staging TNM	Risk factor	I-131	Follow-up time	Evolution
2011	14 y 3 m	M	PTC	Medulloblastoma hypothyroidism	TT	T1aN0M0	Low	Yes	34 m	Remission
2011	14 y 9 m	F	PTC	Hodgkin Lymphoma	TT, LR	T3N1aM0	Intermediary	Yes	80 m	Remission
2011	13 y 4 m	F	PTC	–	TT, LR	T3N1bM0	High	Yes	28 m	Remission
2011	8 y	F	PTC	–	TT, LR	T2N0M0	Low	Yes	51 m	Remission
2012	7 y 10 m	M	PTC	–	TT, LR	T1bN0M0	Low	Yes	71 m	Remission
2013	10 y 10 m	M	PTC	Hashimoto thyroiditis	TT, LR	T1N1aM0	Intermediary	Yes	97 m	Remission
2015	5 y 10 m	F	MTC	RET mutation	TT	T1aN0M0	High	No	31 m	Remission
2015	6 y 4 m	F	PTC	–	TT, LR	T2N1aM0	Intermediary	Yes	67 m	Remission
2015	14 y 6 m	M	FTC	Hodgkin Lymphoma	TT	T2N0M0	Low	No	42 m	Remission
2016	8 y 9 m	F	PTC	Hashimoto thyroiditis	TT, LR	T2N1aM0	Intermediary	Yes	65 m	Without remission
2016	11 y	M	PTC	–	TT, LR	T3N1bM1	High	Yes	65 m	Without remission
2016	13 y 3 m	F	PTC	–	TT, LR	T2N1aM0	Intermediary	Yes	36 m	Remission
2018	5 y	M	MTC	RET mutation	TT	T1aN0M0	High	No	28 m	Remission
2019	10 y 5 m	F	PTC	–	TT, LR	T2N1bM0	High	Yes	28 m	Without remission
2019	12 y 4 m	F	PTC	–	TT, LR	T1bN1bM0	High	Yes	29 m	Without remission
2020	13 y 1 m	F	PTC	Hodgkin Lymphoma	TT	T1aN0M0	Low	No	9 m	Remission

Y: years; TT: total thyroidectomy; m: months; LR: lymph node resection; M: male; I-131: radioiodine therapy with I-131; F: female; PTC: papillary thyroid carcinoma; MTC: medullary thyroid carcinoma; FTC: follicular thyroid carcinoma; TNM: Tumor-Node-Metastasis.

PTC corresponded to 81.25% of cases. Follicular thyroid carcinoma (FTC) occurred in one case, and MTC was present in the form of microcarcinoma in two patients with the RET mutation, who underwent prophylactic TT (Table 1).

Cervical lymph nodes were affected in 56.25%. Only one case had lung metastasis, evidenced by a whole-body scan (WBS) at the time of diagnosis.

According to the ATA stratification, 37.5% of cases were classified as high risk, three of whom had no remission of the disease at the end of the follow-up period. Of the five patients with intermediate risk, four were in remission, and all low-risk patients were in remission at the last follow-up visit (Table 1). Four patients (25%) were classified as high risk, and three had persistent disease from the start of treatment, never achieving remission, with persistently positive Tg or rising anti-Tg antibody levels. One case that presented lung metastasis at diagnosis will undergo another session of I-131, as evidence of the disease remains.

I-131 was used in 75% of differentiated thyroid cancer (DTC) cases (Table 1).

A whole-body diagnostic scan after treatment revealed iodine uptake in eight cases.

During follow-up, one patient with intermediate-risk PTC presented recurrence in the cervical lymph node, requiring additional surgery, and is currently in remission.

The average clinical follow-up time was 47.5 months. No patients died during follow-up.

## DISCUSSION

Pediatric DTC is a rare disease, and published data from retrospective cohorts are scarce. However, its incidence has been growing annually by about 3.74%, explained both by a real increase and by the greater availability and sensitivity of ultrasound, frequent surveillance, and greater access to fine needle aspiration (FNA)^8,9^.

Hypotheses proposed for a true increase in the incidence of DTC include the growing prevalence of pediatric obesity, exposure to sources of environmental radiation, and the increased survival of pediatric cancer patients who have undergone radiation therapy^10–12^.

In the pediatric population, DTC occurs preferentially in adolescents and females, with an incidence 3–5 times higher^1,3^. In this study, there was a predominance of adolescents and females, but with a female/male ratio of 1.7:1, lower than that described in previous studies³, possibly due to the sample size.

Regarding risk factors for PTC, the main isolated factor is thyroid exposure to radiation^6^. Therefore, the medical history of a patient should always include questions about radiation exposure to the head and neck region. It is also important to evaluate the thyroid during physical examinations of all children^1,3^.

According to the ATA^3^, there is no consensus on requesting imaging tests to screen for these cases. However, some authors recommend surveillance with ultrasound in an attempt to detect DTC at an early stage, arguing that early detection can lead to better results and avoid more aggressive treatments^5^.

In this series, four patients with a history of previous malignancy and exposure to cervical radiotherapy were diagnosed with PTC, with the discovery in three cases occurring during routine ultrasound examination in asymptomatic patients, highlighting the importance of ultrasound screening in individuals with risk factors.

Other risk factors for developing DTC include autoimmune thyroid disease, iodine deficiency or excess, and genetic syndromes such as MEN 2. There is some evidence in the literature demonstrating that Hashimoto's thyroiditis is associated with an increased risk of developing thyroid nodules and PTC^1,3^. The frequency of malignancy in pediatric patients with Hashimoto's thyroiditis is described as ranging from 0.67 to 3%, which is above the risk in the general pediatric population, which is approximately 0.02%^12^. Proposed mechanisms for this association include TSH overproduction or chronic inflammation resulting in proliferation, angiogenesis, and/or reduced apoptosis^3^. In the present study, two patients with PTC had Hashimoto's thyroiditis, and two with MTC had a family history of MEN.

In children and adolescents, the most common histological type is PTC, accounting for 80–90% of all cases, followed by FTC (~10%) and MTC (3–5%)^1,3,5,11^. In this series, consistent with the literature^1,3,5,11^, PTC occurred in 81.25% of cases.

Lymph node involvement is more common in PTC, while FTC presents a higher risk of early hematogenous spread to the lungs and bones. The lungs are the most frequent site of metastasis in DTC, affecting about 25% of cases. Compared to adults, children and adolescents have a higher chance of lymph node involvement and distant metastases at diagnosis; however, this does not represent a worse prognosis, suggesting that biological factors and response to treatment are different, thus emphasizing the need for specific recommendations for the pediatric population^3,4^. In the present study (Table 1), of the 13 patients with PTC, 69.23% had lymph node involvement at diagnosis, and one had lung metastasis. The only case of FTC in the series had undergone radiation in the head and neck region for the treatment of Hodgkin's lymphoma and had the disease confined to the gland.

The majority of MTCs in the pediatric population are hereditary, resulting from a pathogenic RET gene variant (codon 634, p.Cys634Arg) present in MEN types 2A and 2B^3,11,12^. MTC is associated with poorer survival due to the bilateral and multifocal nature of the tumor. Therefore, in familial cases, such as MEN 2, prophylactic TT is recommended^3,11^. In this series, the two cases with MTC had a family history of MEN 2, and pathogenic RET gene variant testing was positive, leading to the indication of prophylactic TT.

The most common clinical manifestation of DTC in children and adolescents is thyroid nodules^1,3,5,11,12^. The presence of nodules associated with cervical lymphadenopathy is predictive of malignancy^1,6,13^.

In this study (Figure 1), more than half of the patients (56.25%) had a thyroid nodule on physical examination, which was investigated with ultrasound, followed by FNA^12^. The three patients with previous radiotherapy and the two with Hashimoto's thyroiditis were asymptomatic, with the diagnosis suggested by ultrasound.

The therapeutic guidelines recommended by the ATA advocate an individualized risk-based approach, combining histopathological findings and postoperative clinical data to identify patients who are likely to benefit from additional therapies^3,13^.

In the past, lobectomy or subtotal thyroidectomy was relatively a common approach in children diagnosed with PTC. Long-term analyses have shown that TT reduces the chances of recurrence from 35 to 6% compared to lobectomy, in addition to allowing Tg to be used as a marker to identify persistence or recurrence of the disease^2,3^. However, American and European guidelines suggest that for low-risk patients, lobectomy could be considered to reduce the risk of adverse events without compromising the effectiveness of treatment^3,4^. In the present study, all cases underwent TT (Table 1), regardless of nodule size, considering the increased efficiency of postoperative radioiodine scanning and subsequent ablation. Additionally, multiple studies show that children have an increased incidence of bilateral, multifocal, recorrence, and metastasis^3,6^.

Lymph node dissection was not indicated in two PTC low-risk patients because the tumor was confined to the thyroid, with no clinical or radiologic evidence of nodal involvement or extrathyroidal extension.

In 11 patients in the present study, lymph node resection was associated with malignant cytology and clinical evidence of macroscopic extrathyroid invasion and/or locoregional metastasis in preoperative staging or intraoperative findings.

The most common surgical complications are temporary or permanent hypoparathyroidism, with a rate of 5–15%^3,11,12^. In this study, a quarter of the patients had transient hypoparathyroidism, and one case (T3N1bM1) had hoarseness due to permanent injury to the recurrent laryngeal nerve.

Considering the risk stratification, the ATA^3^ initially recommends performing stimulated Tg in low-risk patients after surgery and, in intermediate- or high-risk groups, indicates performing stimulated Tg in conjunction with a whole body diagnostic scan 12 weeks after TT.

In the past, I-131 was indicated for almost all patients diagnosed with PTC in an attempt to eliminate residual disease and reduce recurrence^1,3^. This universal prescription is no longer encouraged due to possible increases in the rates of secondary malignancies and the lack of evidence demonstrating its benefit in all cases. It is important to consider that, due to the excellent prognosis of PTC in children, these patients are more likely to experience the possible long-term adverse events of I-131 therapy^1,3,9^. However, for patients with lymph node involvement and/or distant metastases, I-131 therapy appears to improve disease-free survival^3,9^.

In this study, the difference in conduct over the years was evident, especially after 2015 with the publication of the ATA guideline^3^, with I-131 therapy not being performed in low-risk patients (Table 1).

Among the 16 patients, four (25%) with papillary thyroid carcinoma, three classified as high risk and one as intermediate risk at diagnosis, had persistent disease and never achieved remission.

DTC has a good prognosis in childhood and adolescence when the correct treatment is performed, despite having a higher chance of metastasis at the time of diagnosis^3,13,14^. In this cohort, a high remission rate of 75% was observed, with no deaths recorded, despite 56.25% of patients presenting cervical lymph node involvement and one of them having pulmonary metastasis at diagnosis, highlighting the effectiveness of the treatment instituted.

This study has limitations related to the sample size, which reduces statistical power, and the fact it was conducted in a single referral center, which may introduce selection bias and limit the generalizability of the results to other populations. Additionally, the observational design prevents causal inferences.

Considering the rarity of the disease in pediatrics, it is essential to conduct a multicenter study, including vulnerable patient groups, in order to increase the number of cases and standardize therapy, in addition to enabling the assessment of the delicate balance between the chance of recurrence and the adverse events of aggressive treatment.

## Data Availability

The datasets generated and/or analyzed during the current study are available from the corresponding author upon reasonable request.
